# Organisational and environmental characteristics of residential aged care units providing highly person-centred care: a cross sectional study

**DOI:** 10.1186/s12912-017-0240-4

**Published:** 2017-08-10

**Authors:** Karin Sjögren, Marie Lindkvist, Per-Olof Sandman, Karin Zingmark, David Edvardsson

**Affiliations:** 1 Department of Nursing, Umeå University, SE-901 87 Umeå, Sweden; 2 Department of Statistics, Umeå School of Business and Economics, Umeå University, Umeå, Sweden; 3 Epidemiology and Global Health, Department of Public Health and Clinical Medicine, Umeå university, Umeå, Sweden; 4 Division of Nursing, Department of Health Science, Luleå University of Technology, Luleå, Sweden; 5 Region of Norrbotten, Luleå, Sweden; 6 Division of Caring Sciences, Department of Neurobiology, Care Sciences and Society (NVS), Karolinska Institutet, Stockholm, Sweden; 7 School of Nursing and Midwifery, La Trobe University, Melbourne, Australia

**Keywords:** Care philosophy, Facilitators, Leadership, Person-Centred Care, Physical environment, Residential care facilities, Social support

## Abstract

**Background:**

Few studies have empirically investigated factors that define residential aged care units that are perceived as being highly person-centred. The purpose of this study was to explore factors characterising residential aged care units perceived as being highly person-centred, with a focus on organisational and environmental variables, as well as residents’ and staff’ characteristics.

**Methods:**

A cross-sectional design was used. Residents (*n* = 1460) and staff (*n* = 1213) data from 151 residential care units were collected, as well as data relating to characteristics of the organisation and environment, and data measuring degree of person-centred care. Participating staff provided self-reported data and conducted proxy ratings on residents*.* Descriptive and comparative statistics, independent samples t-test, Chi^2^ test, Eta Squared and Phi coefficient were used to analyse data.

**Results:**

Highly person-centred residential aged care units were characterized by having a shared philosophy of care, a satisfactory leadership, interdisciplinary collaboration and social support from colleagues and leaders, a dementia-friendly physical environment, staff having time to spend with residents, and a smaller unit size. Residential aged care units with higher levels of person-centred care had a higher proportion of staff with continuing education in dementia care, and a higher proportion of staff receiving regular supervision, compared to units with lower levels of person-centred care.

**Conclusions:**

It is important to target organisational and environmental factors, such as a shared philosophy of care, staff use of time, the physical environment, interdisciplinary support, and support from leaders and colleagues, to improve person-centred care in residential care units. Managers and leaders seeking to facilitate person-centred care in daily practice need to consider their own role in supporting, encouraging, and supervising staff.

## Background

The proportion of old people in the population is increasing in Sweden, and in other western countries. With older age the prevalence of disabilities and chronic diseases, such as dementia increases and consequently also the need for care [1]. This imposes challenges for policy-makers, managers and nursing staff, how to provide care that promotes experiences of well-being, satisfaction, and a meaningful life, in addition to providing merely the symptom control and safe containment of old people and people with dementia.

Research has indicated that residential aged care is at risk of being more task-focused than person-centred [2, 3], that there often is a lack of individually tailored meaningful activities [4–6], and few possibilities for resident choice in daily care and activities [4, 5, 7, 8]. It has also been demonstrated that residents and their family members regard independence, autonomy, and community as important, while staff regard these as less valuable [9]. Thus, there is a need for improvement, and person-centred care is increasingly advocated as a model of care that supports holistic well-being for older people and people with dementia in residential aged care [10–12].

There is a growing body of evidence that different forms of PCC-interventions have positive effect on residents’ well-being and quality of life (QoL) [13–16], levels of depression [13] and agitation [14, 17, 18], mostly investigated in relation to people with dementia living in residential care facilities. It has also been reported positive effects on staff job satisfaction [19, 20], work related stress [20, 21], and burnout [22, 23] from providing person-centred care. However, the evidence to date is not conclusive [24–26], as PCC-studies also report a lack of effect on resident QoL [17, 27], as well as a lack of effect on residents agitation [27–29]. Thus, there seems to be a need for further exploration of factors facilitating successful implementation of PCC.

Reported experiences from culture change programmes and interventions in residential aged care facilities, indicate that factors such as effective collaboration and communication, a shared vison of care philosophy, a supportive and enabling management style, informal and formal supervision influence the extent to which person-centred care is really improved in practice [21, 30–35]. A number of authors argue that development of person-centred care in practice should be based on a multilevel approach including intervention actions related to organisation, environment as well as interventions aiming to develop the attitude and knowledge of staff [36–39]. However, a literature review of PCC-interventions [24] indicated that there remain needs to further explore which personal, organisational, and environmental factors that are required to successfully implement PCC.

In theory, person-centred care has been related to a number of factors such as the cognitive and functional abilities of residents [11], as well as to staff’s professional and interpersonal competence, commitment and self-knowledge [40]. In addition, theory suggests that having a beneficial psychosocial and physical environment, a supportive and present leadership, and a flexible and supportive organisation can enable person-centred processes [40]. Few studies have empirically investigated which factors that defines residential aged care units that are perceived as being person-centred. Among the few studies that exist, S Caspar, HA Cooke, N O’Rourke and SW MacDonald [41] showed in a cross-sectional study in long term care facilities in Canada, that high quality of inter-professional relationships, support and appreciation from management and necessary time positively predicted a perceived ability to provide person-centred care. A few other studies have reported that person-centred units incorporate psychosocial approaches to behavioural symptoms based on interactional and environmental interpretations, environmental adaptations and reflective individualised social interactions [42, 43]. It has also been reported that person-centred care is less related to the length of work experience and formal education of staff and leaders [41, 42], but rather to a focus on social care and everyday activities, staff influence and autonomy [44].

To conclude, there is limited research evidence on which factors actually characterise those residential aged care units perceived as offering more person-centred care. Generating valid and reliable knowledge about such factors appear immensely important in guiding practice improvements and development as well as guiding further research and education on this issue. Thus, there is a need to explore personal, organisational and environmental factors that can characterise person-centred residential aged care units, and this study is an attempt to address that gap in knowledge.

## Methods

### Aim

The aim of this study was to explore factors characterising residential aged care units perceived as being highly person-centred, with a focus on organisational and environmental variables, as well as residents’ and staff’ variables.

### Design

This study had a cross sectional explorative design. The study is part of a larger project that aimed to explore person-centred care in Swedish residential aged care units, and its association with residents’ well-being, staff’ perception of their work environment, and organisational factors. Results focusing on residents well-being and staff work environment have been reported elsewhere [45, 46].

### Sampling and settings

Executive officers of municipality-based elderly care, together with dementia care nurses in a nationwide network for dementia care in Sweden, were invited to participate in recruiting residential aged care units for the study. All dementia care nurses and executive officers that were willing to participate received both oral and written information about the study. A contact person was recruited in each unit to oversee the distribution and collection of study surveys.

A total of 87 residential care facilities participated, amounting to 151 individual care units, as facilities could include several autonomous units. The majority of participating care units were special care units for people with dementia (70%), located in rural (26%) and urban (74%) regions of Sweden. The unit size ranged from four to 26 residents, and between four and 20 staff members worked on a permanent basis within these units. All residents living in the units were included in the study. Staff were included if they were in permanent employment or on fixed-term contracts.

### Data collection

Data were collected through a resident and a staff survey. The resident survey consisted of demographic variables and established measurement scales on ADL-abilities and cognition. Due to a high expected prevalence of cognitive impairment, proxy ratings were performed. This meant that the staff member who knew each resident best was asked to perform the ratings. The staff survey included demographic variables and established instruments for assessing person-centred care of the unit, perceived social support, as well as some study-specific variables. A total of 1655 resident surveys were distributed, and 1471 were returned (89%). Eleven resident surveys were excluded due to multiple missing data (*n* = 1460). A total of 1482 staff surveys were distributed, and 1237 were returned (83%). Twenty-four staff surveys were excluded due to missing data (*n* = 1213). All data was collected in May 2010.

Perceived Person-centred care of the units (PCC) was investigated with the Person-centred Care Assessment Tool (P-CAT) [47, 48]; a scale that measures the extent to which staff perceive care provided as being person-centred. The P-CAT consists of 13 items formulated as statements about the content of care, aspects of the environment and the organisation. A total score is calculated and higher values indicate a higher degree of person-centred care, in a possible range of 13–65. The P-CAT has shown satisfactory estimates of reliability and construct validity in an Australian sample (Cronbach’s α = 0.84) [47] and in a Swedish sample (Cronbach’s α = 0.75) [48].

To investigate residents’ ADL ability five items in the Multi-dimensional Dementia Assessment Scale (MDDAS) [49] was used. Higher scores indicate higher ADL ability in a possible range of 4–24. The prevalence of cognitive capacity was investigated using the Geriatric Rating Scale (GRS) [50], which consists of 27 items concerning the person’s ability to orientate. Higher scores indicate a higher ability to orientate in a possible range of 0–27. Established cut off scores were used to define cognitive impairment or not. A score of less than 24 is considered to indicate cognitive impairment, which correlates with a sensitivity of 90% and a specificity of 91% to the cut-off point 24/30 traditionally used in the Mini-mental State Examination (MMSE) [49].

Factors related to the organisation and the environment were investigated by study-specific variables, and validated scales. Five study-specific variables rated on a 100 mm VAS scale were included; *The physical environment is dementia-friendly, We have an interdisciplinary collaboration in care*, *We have a satisfactory leadership, We have time to spend together with residents, We have a shared philosophy of care*. Higher values implied that staff agreed with the statement. Unit size was investigated by reporting the number of residents living in the unit. Whether or not staff received systematic supervision was investigated with a dichotomous item ‘yes/no’. The subscale social support of the Swedish Demand-Control-Support Questionnaire (DCSQ), [51, 52] was used to investigate the extent to which staff experienced support from co-workers and leaders. The subscale social support consists of 6 items (range of 6–24); a total score was calculated and higher values indicates higher social support. Construct validity and reliability estimates has been reported as satisfactory for the DCSQ (Cronbach’s α = 0.83) [52].

Resident characteristics were investigated by study-specific demographic variables: age; gender; and length of stay in the facility. Staff characteristics were measured by demographic variables: age; gender; years of experience in aged care and work in the current unit, and one dichotomous item regarding continuing education in dementia care.

### Analyses

The IBM SPSS Statistics version 22 ® (SPSS. Release 22. Chicago: SPSS Inc.; 2013) was used to analyse the data. The analysis procedure was performed in several steps. First, sample characteristics were explored using descriptive and comparative statistics. Secondly, PCC was aggregated on unit level (mean value for the unit), and divided into two groups; higher PCC including care units in the highest median (*n* = 74, PCC scores ≥48.79) and lower PCC including care units in the lower median (*n* = 77, PCC scores ≤48.80). Thirdly, differences in resident characteristics, staff characteristics, environmental and organizational factors between units with higher and lower levels of PCC were explored using independent samples t-test for continuous variables and Chi^2^ test for ordinal and nominal variables. Eta Squared and Phi coefficient were used to calculate effect size.

## Results

### Description of the sample

The mean age of residents (*n* = 1460) was 85 years (SD ± 8), and their mean stay in the care unit was about 2.6 years (SD ± 3). The majority were women (70%) and 88% had cognitive impairment. Ten per cent of residents could dress independently, 5 % could manage their personal hygiene independently, and 49% could eat and drink independently. The mean age of staff (*n* = 1213) was 46 years (SD ± 11), they had worked in aged care for about 16 years (SD ± 10) and in the same unit for about 7.6 years (SD ±7). The majority were women (95%), qualified as enrolled nurses (80%), and a slight majority (56%) had some continuing education in dementia care. The sample characteristics of residents and staff in included care units are shown in Tables 1 and 2.

**Table 1:** Characteristics of the sample (residents)

	n^a^ (%)	m (SD)
Sex		
Men	375 (30.1)	
Women	870 (69.9)	
Age, years		85.0 (7.5)
Time in facility, years		2.6 (2.5)
Cognitive impairment		
Yes	1261 (88.3)	
No	67 (11.7)	
Normal ability to dress on own initiative	123 (9.8)	
Manages personal hygiene on own initiative	63 (5.0)	
Eats and drinks independently	611 (48.9)	

^a^n does not always add up to 1460 in all variables due to missing items

**Table 2:** Characteristics of the sample (staff)

	n^a^ (%)	m (±SD)
Sex		
Men	56 (4.8)	
Women	1105 (95.2	
Age (Years)		45.8 (11.2)
Years of experience in aged care		15.9 (10.4)
Years of work in this facility		7.6 (6.9)
Qualifications		
Registered nurses	35 (3.1)	
Enrolled nurses	910 (80.1)	
Nurses’ assistants	120 (10.6)	
No formal qualifications	42 (3.7)	
Other education	29 (2.6)	
Regular supervision	234 (20.9)	
Additional education in dementia care	598 (56.0)	

^a^n does not always add up to 1213 in all variables due to missing items

There were no significant differences related to residents’ characteristics (gender, aged, cognitive impairment, ADL-capacity, length of stay in facility) between units with high PCC (PCC ≥ 48.79) and units with low PCC (≤ 48.80) (see Table 3). For staff characteristics, it was shown that in units with high PCC a higher proportion of staff (61.2%) had some additional education in dementia care compared to units with low PCC (51.1%, *p =* 0.001, *phi* − 0.1). No significant differences were found related to other staff characteristics (gender, aged, experience from aged care).

**Table 3:** Comparison of residents and staff characteristics between units with high and low levels of PCC

	Low PCC		High PCC			
	M(sd)	n(%)	M(sd)	n(%)	*p-value*	*Effect size*
Residents						
Gender						
Women		518 (69.2)		467 (67.3)		
Men		231 (30.8)		227 (32.7)	0.45	
Age	84.5 (3.3)		84.9 (2.8)		0.40	
Cognitive impairment			644 (87.6)	617 (89.0)	0.41	
ADL-ability	13.9 (6.1)		14.3 (6.0)		0.20	
Time for stay in this facility (months)	32.1 (29.7)		31.0 (31.4)		0.50	
Staff						
Gender						
Women		580 (94.2)		564 (96.1)		
Men		36 (5.8)		23 (3.9)	0.10	
Age	45.9 (11.5)		45.7 (10.9)		0.75	
Years of experience in aged care	15.5 (10.4)		16.0 (10.3)		0.39	
Years of work in this facility	7.3 (6.5)		7.9 (7.4)		0.10	
Additional education in dementia care		291 (51.1)		328 (61.2)	0.001	−0.1^a^

^a^phi coefficient

When comparing organisational and environmental variables, it was found that, in units with higher levels of PCC, the number of residents per unit were lower (13.7 ± 9.7) compared to in units with lower PCC (14.8 ± 9.3, *p* = 0.05, *ŋ* = 0.003). The variables interdisciplinary collaboration (*p* < 0.001, *ŋ* = 0.041), satisfactory leadership (*p* < 0.001, *ŋ* = 0.107), a dementia-friendly physical environment, (*p* < 0.001, *ŋ* = 0.045), time to spend with residents (*p* < 0.001, *ŋ* = 0.108), a shared philosophy of care (*p* < 0.001, *ŋ* = 0.090), and social support (*p* < 0.001, *ŋ* = 0.069), were rated significantly higher in units with higher PCC compared to units with lower PCC. In addition, the number of staff who received regular supervision was higher in units with high levels of PCC compared to in units with low levels of PCC (*p* = 0.005, *phi* − 0.09) (Table 4).

**Table 4:** Comparison of organisational and environmental factors between units with high and low levels of PCC

	Low PCC		High PCC			
	M (sd)	N (%)	M (sd)	N (%)	*p*-value	Effect size
Unit size (number of residents)	14.8 (9.3)		13.7 (9.7)		0.05	0.003^a^
Interdisciplinary collaboration	69.0 (28.5)		79.7 (23.6)		< 0.001	0.041^a^
Leadership	60.2 (27.3)		77.1 (21.6)		< 0.001	0.107^a^
Dementia friendly physical environment	50.2 (29.8)		63.1 (29.9)		< 0.001	0.045^a^
Time to spend being with residents	53.6 (27.5)		71.8 (24.7)		< 0.001	0.108^a^
Shared philosophy of care	70.0 (24.8)		83.7 (18.3)		< 0.001	0.090^a^
Social support	19.2 (3.0)		20.7 (2.6)		< 0.001	0.069^a^
Regular supervision		102 (17.4)		139 (24.3)	0.005	−0.09^b^

^a^Eta squared,^b^ Phi coefficient

In this sample, the Cronbach alpha estimates were 0.75 for the P-CAT, 0.84 for the Social support subscale, and 0.95 for the GRS.

## Discussion

The purpose of this study was to explore which factors characterise residential aged care units perceived as being highly person-centred. The results indicate that highly person-centred residential aged care units were characterized by a number of organisational and environmental facilitating factors, i.e. *a shared philosophy of care, time to spend with residents, social support from colleagues and leaders, a dementia-friendly physical environment, interdisciplinary collaboration, regular supervision of staff, and a smaller unit size,* one staff characteristic; *continuing* education in dementia care and no particular resident characteristics.

A shared philosophy of care characterises highly person-centred residential aged care units. This finding confirms the theoretical proposal [37] that a strong shared value base is an important prerequisite for person-centred care, implying that leaders and managers should work with their teams to develop a shared vision of person-centred care [40]. This finding is also supported by experiences from PCC intervention studies and practise development programmes, in which managers and staff describe that the values and ideas of person-centred care need to be embedded in the whole organisation in order to make person-centred care happen [31–33, 53]. To our knowledge, the influence of having a shared philosophy of care as rated by direct care staff, has not been operationalized and studied before in relation to PCC. In a study from the U.S, J Banaszak-Holl, NG Castle, M Lin and G Spreitzer [54] found that nursing homes involved in person-centred development programmes did not report higher person-centred cultural values than other nursing homes. However, as no objective measurement of person-centered care was conducted in the nursing homes included in their study, the impact of cultural values on person-centred care remain unknown. Further studies that explore the content and process in creating shared person-centred care values in nursing homes would be valuable. Questions such as how to facilitate shared values of person-centred care and how such core values are translated into actual day-to-day interactions, priorities and activities need further study.

The results indicate that perceptions of having time as a staff member to just be together with residents was a factor that characterized person-centred residential aged care units. This aspect could be a part of the shared values of PCC, but it can also be a matter of perceived workload if staff perceive themselves as having sufficient time or freedom to prioritise spending time just being with residents. Although it has been reported that staff perceive that a person-centred approach in care takes more time and resources [41], it has also been reported that person-centred care is associated with a better use of time [55, 56], creating more possibilities for positive interactions and encounters.

Few studies have systematically investigated time use in PCC-interventions [16, 17]. L Chenoweth, MT King, YH Jeon, H Brodaty, J Stein-Parbury, R Norman, M Haas and G Luscombe [17] reported the cost for implementation of two PCC-models (Dementia Care Mapping and PCC-education), which included time for education and training and the time staff used on PCC activities that were related to the intervention. It was demonstrated that education in PCC was more cost effective than using Dementia Care Mapping in relation to reduction in agitation scores, and there was a decreased cost per unit reduction in agitation scores over time for PCC-education. As agitation is distressing and drives cost for increased staff time, the results of the study [17] may be interpreted to indicate that PCC may take additional time initially, with subsequent time and cost savings in the long term.

Another aspect, reported in intervention studies, is that care staff describe that competing demands in addition to lack of time is a barrier to PCC [30]. This highlights the importance for managers and leaders to support direct care workers in making priorities in daily care that facilitates person-centred care. Again this seems related to an overall philosophy of care and if such an explicit or implicit philosophy contains a focus on completing tasks or facilitating well-being.

Social support from colleagues and leaders and satisfactory leadership were also factors related to person-centred care. Theoretical models have previously described the importance of a supportive leader who takes responsibility for an effective, sustainable person-centred culture change and quality improvement in residential aged care facilities [40, 57, 58]. However, few research studies have investigated the importance of leadership and leadership support in enhancing person-centred care. PV Hunter, T Hadjistavropoulos, L Thorpe, LM Lix and DC Malloy [59] showed in an explorative study in two Canadian long-term care homes that structural supervisory and organizational support was not related to different dimensions of PCC, while leaders’ support for collaboration in care was related to different aspects of PCC. In a recently published controlled trial, Y-H Jeon, JM Simpson, Z Li, MM Cunich, TH Thomas, L Chenoweth and HL Kendig [60] reported no change in person-centred care after a 12-month leadership development programme. A Backman, K Sjögren, M Lindkvist, H Lövheim and D Edvardsson [61] reported of positive relationships between effective leadership behaviours and person-centred care. This study contributes to the existing research evidence, by suggesting that the support from managers and leaders is important for facilitating person-centred care.

The results of this study also indicate that a physical environment experienced as dementia-friendly is a factor related to person-centred care. Others have conceptualised a person-centred physical environment as facilitating the independence of residents, positive interactions between residents and staff, and resident participation in collaborative work [62], with the potential to support or hinder residents’ autonomy [59], and allowing residents to be an active participant in everyday life rather than a passive recipient of care [63]. This is supported by results from intervention studies showing that person-centred changes to the environment have had positive effect on QoL and agitation for residents with dementia [16, 64].

The results of this study indicate that the physical environment is an important and meaningful factor in person-centred practice which supports theoretical models proposing that, in order to support person-centred care in residential aged care facilities, it is necessary to change environments to match the competence of the individual [39].

### Methodological considerations

The cross-sectional nature of this study limits causal interpretations of the results. Further studies with interventional and longitudinal designs are needed to further investigate causality. Another limitation is that the study relies on self-reported (staff) data of person-centred care. It is not known whether or not ratings correspond to what actually happens in practice, and thus further studies that combine staff ratings, and family members ratings with observations would be valuable so as to investigate the extent to which behaviours correspond to what is reported. The choice of measurement scales to use when evaluating person-centred care of the unit may need consideration. The P-CAT as used in this study is a short and comprehensive scale including concrete items that are formulated to capture essential elements of person-centred care in units, and it was found appropriate to use as a numerical way to differentiate and compare units with higher and lower degrees of person-centred care. However, it would be valuable to further investigate factors that contribute to define highly person-centred units, including multiple measures of person-centred care to gain further knowledge that can guide PCC-development work. The P-CAT is limited to staff perceptions of person-centred care, and thus other perspectives on this would be valuable.

Nevertheless, the study draws on a large sample from a geographically varied area of Sweden, includes both resident and staff ratings, and makes contemporary relevant analyses of empirical relationships between nursing variables based on extensive nursing theory. Thus, the study makes a small but significant contribution to the literature on the sometimes elusive concept of person-centred care in nursing, by providing empirical data that further contributes to our understanding of what characterises residential aged care units perceived as being person-centred.

A strength of this study is the large sample size of participating units. A network of dementia care nurses in Sweden assisted in recruiting units for this study. In Sweden, dementia care nurses are responsible for dementia care in the municipalities, commonly well-known by staff and managers in residential aged care, and their engagement was instrumental in encouraging facilities and units to participate in the study. Furthermore, unit scores on variables such as person-centred care and social support, were promised and later reported back to the unit together with a short text on how to interpret and understand the scores and results. This reporting back of scores and results is likely to have served as an encouragement to participate in the study, as it provided managers and staff measurable and comparable data as a baseline for their own practice development work, as well as an entry point and incitement to continue with repeated measures and practice development work.

## Conclusions

The knowledge concerning facilitating factors related to person-centred care is mainly based on theoretical discussions and models. This study indicates that highly person-centred residential aged care units can be characterised by a number of organisational and environmental factors, which can be interpreted as facilitators for person-centred care. The study contributes to the emerging research knowledge by suggesting areas that can be assessed and targeted in efforts to facilitate person-centred care in residential aged care units.

It seems important to create a shared philosophy of care among all those involved – managers, care workers, residents and family members – and to enhance care workers in making priorities that increase time and possibilities for social interactions and encounters. Furthermore, changing the physical environment so that it matches residents’ abilities and preferences, and prioritizing support and collaboration between staff members is also important to increase person-centred care in residential care units. Managers and leaders seeking to improve person-centred care will need to consider their own role in supporting, encouraging, and supervising staff in this practice improvement.

## Abbreviations

ADL: Activities of Daily Living
PCC: Person-Centred Care
QoL: Quality of Life
